# Clinical and Genetic Analysis of CHD7 Expands the Genotype and Phenotype of CHARGE Syndrome

**DOI:** 10.3389/fgene.2020.00592

**Published:** 2020-06-18

**Authors:** Zailong Qin, Jiasun Su, Mengting Li, Qi Yang, Shang Yi, Haiyang Zheng, Qiang Zhang, Fei Chen, Sheng Yi, Weiliang Lu, Wei Li, Limei Huang, Jing Xu, Yiping Shen, Jingsi Luo

**Affiliations:** ^1^Genetic and Metabolic Central Laboratory, Guangxi Birth Defects Research and Prevention Institute, Maternal and Child Health Hospital of Guangxi Zhuang Autonomous Region, Nanning, China; ^2^Department of Neonatology, Maternal and Child Health Hospital of Guangxi Zhuang Autonomous Region, Nanning, China; ^3^Department of Genetics, Harvard Medical School, Boston, MA, United States

**Keywords:** CHD7, CHARGE syndrome, dyspnea, neonate, mutation

## Abstract

CHARGE syndrome is a life-threatening disease caused by mutations of chromodomain helicase DNA-binding protein 7 gene (CHD7). The disease is characterized by a pattern of congenital anomalies that involve multiple organs. In this study, five patients were diagnosed as CHARGE syndrome with CHD7 mutations by whole exome sequencing. Although the clinical phenotypes of probands are highly variable and typical symptoms such as coloboma and choanal atresia are not commonly manifested in this cohort, they all presented congenital heart defects. Of note, dyspnea is the most prominent symptom in all five neonatal patients, suggesting that dyspnea might be a phenotypic clue of CHARGE syndrome.

## Introduction

CHARGE syndrome (OMIM#214800), a rare congenital disorder, occurs in approximately 1/8,5000 to 1/15,000 livebirths. It previously described by Hall and Hittner et al. as association of coloboma, choanal atresia, and congenital heart defects and then summarized by Pagon et al. as the acronym of multiple anomalies, including **C**oloboma of eye, **H**eart defects, **A**tresia of choanae, **R**etardation of growth and/or development, **G**enitourinary defects and/or hypogonadism, and **E**ar anomalies with or without deafness (Hall, 1979; Hittner et al., 1979). The clinical diagnostic criteria for CHARGE syndrome were proposed by Blake et al. and Verloes that generally divided into (i) major criteria, including coloboma, choanal atresia/cleft, and hypoplastic semi-circular canals and/or abnormal ears; and (ii) minor criteria, including heart/or esophagus malformation, ear anomalies, rhombencephalic dysfunction, hypothalamic-pituitary deficiency, and intellectual disability (Blake et al., 1998; Verloes, 2005). Moreover, Verloes further classified the CHARGE syndrome into typical CHARGE, partial CHARGE, and atypical CHARGE based on how many characteristics the patient meets (Verloes, 2005). Until 2004, mutations of chromodomain helicase DNA-binding protein 7 (CHD7) was found to be major cause for CHARGE syndrome and appear autosomal dominant inheritance pattern (Vissers et al., 2004). So far, more than 1,000 variants of CHD7 have been identified with next-generation sequencing (NGS), and 90–95% of patients carried a CHD7 variant meet Blake or Verloes’ diagnostic criteria (Jongmans et al., 2006; Bergman et al., 2011). Moreover, most of variants are *de novo*. Therefore, the latest clinical criteria have incorporated pathogenic CHD7 mutations into a major criterion in 2016 (Hale et al., 2016).

CHARGE syndrome causes patients to suffer multiple life-threatening symptoms after birth and brings a lot of burden to their family. However, molecular diagnosis is only made after termination of pregnancy. An efficient strategy for prenatal diagnosis of CHARGE syndrome is urgently needed (Colin et al., 2012).

In our cohort, we have reported five patients diagnosed with CHARGE syndrome and the phenotypes were highly variable. Despite the typical phenotypes sparsely presented, dyspnea was found to be the most prominent symptom in all five patients. Molecular analysis identified five variants of CHD7 including two types of mutations and three of them are novel. Genotype analysis of CHD7 based on ClinVar and CHD7 database demonstrated the mutation spectrum and phenotypes of CHARGE syndrome which might be contributed to the study of CHD7 mutation-associated CHARGE syndrome. Therefore, both reported phenotypes and the mutation spectrum in this study can facilitate the development of prenatal screening for CHARGE syndrome. Our findings may provide new molecular evidence and expand the phenotypes for CHARGE syndrome which would be helpful for clinical diagnosis.

## Materials and Methods

### Sample Collection and DNA Extraction

Genomic DNA was isolated from peripheral blood lymphocytes using Lab-Aid DNA kit (Zeesan Biotech Co., Ltd, Xiamen, China).

### Whole Exome Sequencing and Data Analysis

Genomic DNA was extracted and captured to create the library for whole exome sequencing by Agilent SureSelect Human Exon V6 kit or Agilent SureSelect Clinical Research Exome V2 kit (Agilent Technologies, Santa Clara, CA, United States) according to the manufacturer’s protocol. The libraries were sequenced by HiSeq X Ten or Nova Seq 6000 with PE150 strategy (Illumina, San Diego, CA, United States) with a read depth over 120X and more than 95% of the targeted regions covered over 20X. The sequencing reads were mapped to the Genome Reference Consortium Human genome build 37 (GRCh37). The Genome Analysis Toolkit (GATK) was used for variant calling. TGex software (LifeMap Sciences, Alameda, CA, United States) was used to annotate the variants. Transcript NM_017780.3 was used as the reference sequence. The CHD7 variants and their origins were verified by Sanger sequencing. All the variants were classified according to ACMG/AMP guidelines.

### Editorial Policies and Ethical Considerations

The study was approved by the ethics committees of Maternal and Child Health Hospital of Guangxi Zhuang Autonomous Region. Written informed consent for participation in this study was collected from the family.

## Patients and Clinical Information

In this cohort, five patients including four males and one female were diagnosed by whole exome sequencing. All infants presented dyspnea after birth. Four of them presented different extent of congenital heart defects with symptoms of patent ductus arteriosus (PDA, patients 3, 4, and 5), atrial septal defect (ASD, patients 1, and 5), patent foramen ovale (PFO, patients 4, and 5), and right aortic arch (RAA, patient 1). Three of them presented malformations of either the middle or external ear (patients 1, 2, and 4). Apart from that, macrocephaly (patient 1), microcephaly (patient 2), and ocular coloboma (patients 3, 4, and 5) were also presented.

Specifically, patient 1 was a premature infant with macrocephaly, low-set ears, short neck, ASD, RAA, and micropenis. Both patients 2 and 4 were born with stridor, microtia, and dyspnea. Additionally, patient 2 also had microcephaly with 34 cm (-3 SD) of head circumference and patient 4 suffered PFO, PDA, and retinal coloboma. Patient 3 was a male neonate with severe clinical manifestations who was born by cesarean section due to fetal distress on 37 + 2 w of pregnancy. After his birth, he had no cry and was diagnosed with neonatal pneumonia which lead to a suspicion of congenital airway deformities. Chest CT scan of mediastinal cystic image revealed that he had hiatus hernia. The blood test detected neonatal hypoglycaemia, hypocalcemia, low thyroid stimulating hormone, and lymphopenia. Microcephaly, PDA, abnormal thorax, micropenis, and dysphagia were also presented. Moreover, MRI found anomalies in his bilateral frontal gyrus, sulcus, and middle lobe of cerebellum, as well as widened posterior fossa and cerebellar vermis hypoplasia (CVH) in his brain. Patient 5 was hospitalized for dyspnea and fever and then admitted to neonatal pneumonia. He presented microphthalmia, ASD, PDA, PFO, cardiac insufficiency, optic discs coloboma (ODC), and chorioretinal coloboma, as well as neonatal hyperbilirubinemia and subependymal hemorrhage. The clinical information of patients can be found in Table 1.

**TABLE 1:** Clinical features of patients with CHD7 mutation.

	Patient 1	Patient 2	Patient 3	Patient 4	Patient 5
Variants	c.1480C > *T* p.Arg494*	c.2828_2829delAG p.Glu943fs	c.4667dupC p.Arg1557fs	c.6079C > *T* p.Arg2027*	c.7873C > *T* p.Gln2625*
Inheritance	*De novo* Known	*De novo* Novel	*De novo* Novel	*De novo* Known	*De novo* Novel
Age	13 days	2 months	4 months	12 days	15 days
Gender	Male	Male	Male	Female	Male
Perinatal	Premature birth, Dyspnea	Stridor, Dyspnea	Fetal distress, Dyspnea, Hypoglycemia, Hypocalcemia, Low TSH, Dysphagia	Stridor, Microtia, Dyspnea	Dyspnea, Hyperbilirubinemia, Subependymal hemorrhages
Head & Neck	Low-set ears, Macrocephaly, Short neck	Microtia, Microcephaly	Microcephaly, ODC, Chorioretinal coloboma, Hiatus hernia, CVH, Widened posterior fossa	Retinal coloboma, Microtia	Microphthalmia, ODC, Chorioretinal coloboma
Cardiovascular	ASD, RAA	Normal	PDA	PDA, PFO	ASD, PDA, PFO
Genitourinary	Micropenis	Normal	Micropenis	Normal	Normal
Classification	Pathogenic	Pathogenic	Pathogenic	Pathogenic	Pathogenic

TSH, thyroid stimulating hormone; ODC, Optic disk coloboma; CVH, Cerebellar vermis hypoplasia; PDA, Patent ductus arteriosus; ASD, Atrial septal defect; PFO, Patent foramen ovale; RAA, Right aortic arch; and *, nonsense.

## Molecular Analysis

Data of whole exome sequencing identified five heterozygous null variants of CHD7 in these patients and they were classified to be pathogenic according to ACMG/AMP guidelines. They include (i) two frameshift: c.2828_2829delAG and c.4667dupC; and (ii) three nonsense: c.1480C > *T*, c.6079C > *T*, and c.7873C > *T*. Moreover, except c.1480C > *T* and c.6079C > *T* were reported mutations, the other three were identified as novel mutations in CHD7 (NM_017780.3). Sanger sequencing of parental DNA confirmed that all these variants were *de novo*.

**FIGURE 1:**
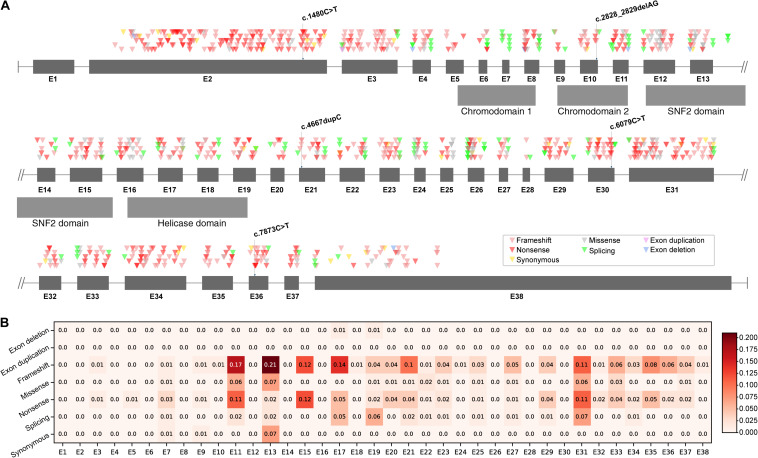
Mutations distribution of CHD7. **(A)** Pathogenic and likely pathogenic variants were collected from ClinVar and CHD7 database and then shown in the schematic according to their chromosomal location. **(B)** The mutation rate of each exon was computed as the quotient between the number of mutations and the number of bases of the exon. Darker color stands for higher mutation rate.

As two types of mutations occurred in five different exons and different domains of CHD7 (Figure 1A), we further raised two questions: (i) Did the observed mutations match with the expected mutations rate of CHD7? (ii) Did the CHD7 genotypes correlate with the CHARGE phenotypes? In order to clarify the mutation spectrum and phenotypes of CHARGE syndrome, we summarized all the pathogenic and likely pathogenic variants from ClinVar and CHD7 database^1^. A total of 925 variants including 375 frameshift (40.54%), 299 nonsense (32.3%), 101 missense (10.92%), 109 splicing (11.78%), 34 synonymous (3.68%), 6 exon deletions (0.65%), and 1 exon duplication (0.11%) were shown in CHD7 exons based on their chromosomal location (Supplementary Table 1). In our cohort, the observed mutation rate of frameshift (2/5, 40%) matched with the expected frameshift mutation rate, the nonsense (3/5, 60%) were more than the expected. To locate the expected mutation type on each exon, frameshift is most frequently detected in E2. Noticed that there are no pathogenic or likely pathogenic variants in E1 and 3′-terminal of E38. Correlation between genotype and phenotype is negative. The exonic mutation rate for each mutation type can be found in Figure 1B. Comparing the incidence of certain type of variants to the exonic mutation rate, there is no significant relevance between the incidence and the type of mutations of CHD7 for our cohort.

## Discussion

CHARGE syndrome is a genetic disorder with highly variable phenotypes even within a family and results from mutations of CHD7 gene. In this study, we report five patients diagnosed as CHARGE syndrome by whole exome sequencing. The phenotypes of patients in this cohort were quite variable. Specifically, the typical features of CHARGE syndrome were dispersedly manifested in patients: (i) **C**oloboma were found in patients 3, 4, and 5, (ii) **H**eart defects were presented in patients 1, 3, 4, and 5, (iii) **A**tresia was not found, (iv) **R**etardation of growth or development needed follow-up examination, (v) **G**enital malformation (micropenis) was only found in patients 1 and 3, and (vi) **E**ar anomalies including microtia and low-set ears were found in patients 1, 2, and 4. Apart from that, premature birth that occurred in patient 1 was also considered to be one of clinical features of CHARGE syndrome (Sanchez et al., 2019). Fetal distress was presented in patient 3 and lead to cesarean section. In addition, the malformations of head and neck were commonly among probands in this cohort. Although the phenotypes are variable in patients with CHARGE syndrome, it is worth noting that dyspnea presented in all five infants after birth which is consistent with previous reports (Daniel, 2008; Verloes, 2005; Xu et al., 2018), suggesting that dyspnea might be considered to be one of clinical diagnosis clues of CHARGE syndrome.

Genetic analysis of CHD7 variants in these patients found that all five variants were *de novo* and were null mutations including two frameshift and three nonsense, leading to loss of function. In addition, three of them were novel and two have been reported in CHARGE patients previously (Janssen et al., 2012; Busa et al., 2016; Moccia et al., 2018). Analysis of mutation spectrum and exon mutation rate in CHD7 (NM_017780.3) revealed that the most common type of mutations is frameshift (375/925, 40.54%), followed by nonsense (299/925, 32.32%). In addition, these frequently detected mutation types are enriched in E2. Surprisingly, there is no record of pathogenic or likely pathogenic variant in exon 1, suggesting that mutations occurring in E1 are probably lethal *in utero*, and the mutations in 3′-terminal of E38 may have no effect on CHD7 protein function due to nonsense mediated decay (Nguyen et al., 2014). Our cohort found two frameshifts (2/5, 40%) occurred in E10 and E21 which matched with the expected mutation rate; three nonsense (3/5, 60%) were found in E2, E30, and E36 which were more than the expected. However, the sample size is very limited and needs to be further explored. Furthermore, to investigate the correlation between certain type of mutation and exonic mutation rate, we calculated the exonic mutation rate for each mutation type (Figure 1B). However, there was no significant correlation between them.

Using genetic testing to predict the risk or confirm a diagnosis of inheritable disease has been extensively implicated. We tried to correlate the genetic mutations of CHD7 to the clinical symptoms of CHARGE patients in this cohort and data from CHD7 database. However, clinical and genetic analysis of CHD7 variants revealed that there are no significant correlations between phenotype and genotype (Supplementary Material). It is consistent with previous report published by Lalani et al. which have analyzed and confirmed that there was no correlation of severity of phenotype with mutations in specific domains of the CHD7 protein (Lalani et al., 2006). And the genotype-phenotype correlations were also confirmed to be negative in three other cohorts (Jongmans et al., 2006; Sohn et al., 2016; Legendre et al., 2017).

Prenatal diagnosis of CHARGE syndrome is still a challenge and mostly depends on the clinical diagnosis with prenatal ultrasound screening. Heart defects, malformations of head and neck such as microcephaly, microtia, and microphthalmia found by ultrasound can be the clues for prenatal diagnosis of CHARGE syndrome. Moreover, fetal *de novo* mutations screening by non-invasive prenatal test (NIPT) with maternal plasma is highly efficient for diagnosis. Detection of mutations in E1 and E38 may also provide clues for predicting severity of CHARGE syndrome by NIPT with maternal plasma.

Despite increasing number of CHD7 variants and associated features of CHARGE syndrome have been reported, the incidence of certain mutations and the effects on the phenotype remain to be illusive. Our study identified three novel variants (c.2828_2829delAG, c.4667dupC, and c.7873C > *T*) and two reported variants (c.4667dupC and c.1480C > *T*) that contributed to CHARGE syndrome. In summary of clinical phenotypes of five cases, our study suggested that postnatal dyspnea can be considered as one of clinical diagnosis clues for CHARGE syndrome, especially when it presents along with typical structural malformations. In conclusion, although there is no significant correlation between genotype and phenotype in this cohort, our study can provide a fundamental understanding and enhance the importance of studying molecular pathology of CHARGE syndrome.

## Data Availability Statement

The BAM files of whole exome sequencing have deposited in BioProject database (https://www.ncbi. nlm.nih.gov/bioproject/604965).

## Ethics Statement

The study was approved by the Ethics Committees of Maternal and Child Health Hospital of Guangxi Zhuang Autonomous Region. Written informed consent to participate in this study was provided by the participants’ legal guardian/next of kin.

## Author Contributions

JS and ZQ conceived and designed the experiments, and wrote the manuscript. QZ, FC, SY, WLu, WLi, and LH performed the experiments. ML, QY, SY, HZ, JX, and YS were involved in data analysis. JL helped to revise the manuscript. All authors read and approved the final manuscript.

## Conflict of Interest

The authors declare that the research was conducted in the absence of any commercial or financial relationships that could be construed as a potential conflict of interest.

## Footnotes

1 www.CHD7.org
