# Supplementary material for: Clinical and Genetic Analysis of CHD7 Expands the Genotype and Phenotype of CHARGE Syndrome
Source: Front Genet. 2020 Jun 18;11:592. doi: 10.3389/fgene.2020.00592 (PMC7314916; doi:10.3389/fgene.2020.00592)
Supplement: Supplementary file 1 [file Data_Sheet_1.zip › Supplementary Table 3 - 6.DOCX]

**Supplemental Materials**

**Supplemental table 3 The occurrence of phenotypes in different mutation types**

| **Mutation type** | **Coloboma** | **Congenital heart defect** | **Choanal anomaly** | **C_L_P** | **Growth retardation** | **Developmental delay** | **Genital hypoplasia** | **External ear anomaly** | **Hearing loss** | **Semicircular canal anomaly** | **Facial palsy** | **TE anomaly** | **Feeding difficulties** | **Other** |
| --- | --- | --- | --- | --- | --- | --- | --- | --- | --- | --- | --- | --- | --- | --- |
| frameshift | 107 | 118 | 63 | 43 | 52 | 21 | 83 | 121 | 110 | 54 | 48 | 23 | 43 | 46 |
| nonsense | 134 | 148 | 75 | 57 | 66 | 23 | 86 | 146 | 118 | 64 | 46 | 28 | 45 | 55 |
| missense | 37 | 31 | 14 | 13 | 19 | 6 | 23 | 47 | 39 | 22 | 9 | 3 | 10 | 14 |
| deletion | 3 | 1 | 1 | 0 | 1 | 0 | 4 | 4 | 4 | 1 | 1 | 0 | 2 | 2 |
| **total** | **281** | **298** | **153** | **113** | **138** | **50** | **196** | **318** | **271** | **141** | **104** | **54** | **100** | **117** |

The values represent the number of occurrence of phenotypes. C_L_P: Cleft lip and/or palate. TE anomaly: Tracheo-esophageal anomaly. Data from CHD7 database.

**Supplemental table 4 The frequency of mutation types in different phenotypes**

| **Mutation type** | **Coloboma** | **Congenital heart defect** | **Choanal anomaly** | **C_L_P** | **Growth retardation** | **Developmental delay** | **Genital hypoplasia** | **External ear anomaly** | **Hearing_loss** | **Semicircular canal anomaly** | **Facial palsy** | **TE anomaly** | **Feeding_difficulties** | **Other** |
| --- | --- | --- | --- | --- | --- | --- | --- | --- | --- | --- | --- | --- | --- | --- |
| frameshift | 0.38 | 0.40 | 0.41 | 0.38 | 0.38 | 0.42 | 0.42 | 0.38 | 0.41 | 0.38 | 0.46 | 0.43 | 0.43 | 0.39 |
| nonsense | 0.48 | 0.50 | 0.49 | 0.50 | 0.48 | 0.46 | 0.44 | 0.46 | 0.44 | 0.45 | 0.44 | 0.52 | 0.45 | 0.47 |
| missense | 0.13 | 0.10 | 0.09 | 0.12 | 0.14 | 0.12 | 0.12 | 0.15 | 0.14 | 0.16 | 0.09 | 0.06 | 0.10 | 0.12 |
| deletion | 0.01 | 0.00 | 0.01 | 0.00 | 0.01 | 0.00 | 0.02 | 0.01 | 0.01 | 0.01 | 0.01 | 0.00 | 0.02 | 0.02 |

The values represent the incidence of mutation types. C_L_P: Cleft lip and/or palate. TE anomaly: Tracheo-esophageal anomaly. Data from CHD7 database.

**Supplemental table 5 The occurrence of phenotypes in different CHD7 domain**

| **Domain** | **Coloboma** | **Congenital heart defect** | **Choanal anomaly** | **C_L_P** | **Growth retardation** | **Developmental delay** | **Genital hypoplasia** | **External ear anomaly** | **Hearing loss** | **Semicircular canal anomaly** | **Facial palsy** | **TE anomaly** | **Feeding difficulties** | **Other** |
| --- | --- | --- | --- | --- | --- | --- | --- | --- | --- | --- | --- | --- | --- | --- |
| BRK1 | 3 | 2 | 2 | 0 | 3 | 1 | 3 | 4 | 4 | 3 | 0 | 0 | 2 | 1 |
| BRK2 | 3 | 4 | 1 | 1 | 2 | 0 | 2 | 3 | 3 | 2 | 2 | 2 | 2 | 1 |
| CD1 | 1 | 1 | 0 | 0 | 1 | 0 | 1 | 1 | 1 | 0 | 0 | 0 | 0 | 1 |
| Chromodomain 1 | 12 | 17 | 8 | 4 | 6 | 4 | 10 | 18 | 12 | 6 | 3 | 2 | 2 | 6 |
| Chromodomain 2 | 2 | 3 | 2 | 1 | 1 | 0 | 2 | 3 | 2 | 2 | 2 | 0 | 1 | 2 |
| DEAD like helicase superfamily | 0 | 0 | 0 | 0 | 0 | 0 | 0 | 0 | 0 | 0 | 0 | 0 | 0 | 0 |
| HD | 1 | 1 | 2 | 0 | 2 | 0 | 0 | 1 | 1 | 0 | 0 | 0 | 0 | 3 |
| Helicase superfamily C-terminal domain | 18 | 14 | 8 | 8 | 7 | 2 | 10 | 18 | 15 | 8 | 5 | 2 | 4 | 1 |
| SANT | 0 | 1 | 1 | 0 | 1 | 0 | 1 | 1 | 1 | 0 | 0 | 0 | 0 | 1 |
| SANT DNA binding | 9 | 9 | 5 | 3 | 5 | 3 | 8 | 11 | 1 | 5 | 4 | 4 | 4 | 5 |
| SNF2 family N-terminal domain | 7 | 6 | 2 | 5 | 4 | 2 | 5 | 10 | 9 | 5 | 1 | 1 | 3 | 3 |
| SNF2 family N-terminal domain and DEAD-like helicase superfamily | 1 | 1 | 1 | 0 | 1 | 0 | 1 | 0 | 0 | 0 | 0 | 0 | 0 | 0 |
| SNF2 family N-terminal domain and DEAD-like helicase superfamily and helicase ATP binding | 18 | 19 | 9 | 6 | 7 | 5 | 18 | 20 | 21 | 10 | 5 | 1 | 6 | 7 |
| Non | 206 | 220 | 112 | 85 | 98 | 33 | 135 | 228 | 193 | 100 | 82 | 42 | 76 | 86 |
| **total** | **281** | **298** | **153** | **113** | **138** | **50** | **196** | **318** | **263** | **141** | **104** | **54** | **100** | **117** |

The values represent the number of occurrence of phenotypes. C_L_P: Cleft lip and/or palate. TE anomaly: Tracheo-esophageal anomaly. Data from CHD7 database.

**Supplemental table 6 The frequency of CHD7 domain mutation in different phenotypes**

| **Domain** | **Coloboma** | **Congenital heart defect** | **Choanal anomaly** | **C_L_P** | **Growth retardation** | **Developmental delay** | **Genital hypoplasia** | **External ear anomaly** | **Hearing loss** | **Semicircular canal anomaly** | **Facial palsy** | **TE anomaly** | **Feeding difficulties** | **Other** |
| --- | --- | --- | --- | --- | --- | --- | --- | --- | --- | --- | --- | --- | --- | --- |
| BRK1 | 0.01 | 0.01 | 0.01 | 0.00 | 0.02 | 0.02 | 0.02 | 0.01 | 0.02 | 0.02 | 0.00 | 0.00 | 0.02 | 0.01 |
| BRK2 | 0.01 | 0.01 | 0.01 | 0.01 | 0.01 | 0.00 | 0.01 | 0.01 | 0.01 | 0.01 | 0.02 | 0.04 | 0.02 | 0.01 |
| CD1 | 0.00 | 0.00 | 0.00 | 0.00 | 0.01 | 0.00 | 0.01 | 0.00 | 0.00 | 0.00 | 0.00 | 0.00 | 0.00 | 0.01 |
| Chromodomain 1 | 0.04 | 0.06 | 0.05 | 0.04 | 0.04 | 0.08 | 0.05 | 0.06 | 0.05 | 0.04 | 0.03 | 0.04 | 0.02 | 0.05 |
| Chromodomain 2 | 0.01 | 0.01 | 0.01 | 0.01 | 0.01 | 0.00 | 0.01 | 0.01 | 0.01 | 0.01 | 0.02 | 0.00 | 0.01 | 0.02 |
| DEAD like helicase superfamily | 0.00 | 0.00 | 0.00 | 0.00 | 0.00 | 0.00 | 0.00 | 0.00 | 0.00 | 0.00 | 0.00 | 0.00 | 0.00 | 0.00 |
| HD | 0.00 | 0.00 | 0.01 | 0.00 | 0.01 | 0.00 | 0.00 | 0.00 | 0.00 | 0.00 | 0.00 | 0.00 | 0.00 | 0.03 |
| Helicase superfamily C-terminal domain | 0.06 | 0.05 | 0.05 | 0.07 | 0.05 | 0.04 | 0.05 | 0.06 | 0.06 | 0.06 | 0.05 | 0.04 | 0.04 | 0.01 |
| SANT | 0.00 | 0.00 | 0.01 | 0.00 | 0.01 | 0.00 | 0.01 | 0.00 | 0.00 | 0.00 | 0.00 | 0.00 | 0.00 | 0.01 |
| SANT DNA binding | 0.03 | 0.03 | 0.03 | 0.03 | 0.04 | 0.06 | 0.04 | 0.03 | 0.00 | 0.04 | 0.04 | 0.07 | 0.04 | 0.04 |
| SNF2 family N-terminal domain | 0.02 | 0.02 | 0.01 | 0.04 | 0.03 | 0.04 | 0.03 | 0.03 | 0.03 | 0.04 | 0.01 | 0.02 | 0.03 | 0.03 |
| SNF2 family N-terminal domain and DEAD-like helicase superfamily | 0.00 | 0.00 | 0.01 | 0.00 | 0.01 | 0.00 | 0.01 | 0.00 | 0.00 | 0.00 | 0.00 | 0.00 | 0.00 | 0.00 |
| SNF2 family N-terminal domain and DEAD-like helicase superfamily and helicase ATP binding | 0.06 | 0.06 | 0.06 | 0.05 | 0.05 | 0.10 | 0.09 | 0.06 | 0.08 | 0.07 | 0.05 | 0.02 | 0.06 | 0.06 |
| Non | 0.73 | 0.74 | 0.73 | 0.75 | 0.71 | 0.66 | 0.69 | 0.72 | 0.73 | 0.71 | 0.79 | 0.78 | 0.76 | 0.74 |

The values represent the mutation rate in different domain. C_L_P: Cleft lip and/or palate. TE anomaly: Tracheo-esophageal anomaly. Data from CHD7 database.
